# Stress can be detected during emotion-evoking smartphone use: a pilot study using machine learning

**DOI:** 10.3389/fdgth.2025.1578917

**Published:** 2025-04-30

**Authors:** Lydia Helene Rupp, Akash Kumar, Misha Sadeghi, Lena Schindler-Gmelch, Marie Keinert, Bjoern M. Eskofier, Matthias Berking

**Affiliations:** ^1^ Lehrstuhl für Klinische Psychologie und Psychotherapie, Friedrich-Alexander-Universität Erlangen-Nürnberg, Erlangen, Germany; ^2^ Machine Learning and Data Analytics Lab, Faculty of Engineering, Friedrich-Alexander-University Erlangen-Nürnberg, Erlangen, Germany; ^3^ Translational Digital Health Group, Institute of AI for Health, Helmholtz Zentrum München - German Research Center for Environmental Health, Friedrich-Alexander-Universität Erlangen-Nürnberg, Erlangen, Germany

**Keywords:** stress, emotion, machine learning, emotion expression, automated stress recognition

## Abstract

**Introduction:**

The detrimental consequences of stress highlight the need for precise stress detection, as this offers a window for timely intervention. However, both objective and subjective measurements suffer from validity limitations. Contactless sensing technologies using machine learning methods present a potential alternative and could be used to estimate stress from externally visible physiological changes, such as emotional facial expressions. Although previous studies were able to classify stress from emotional expressions with accuracies of up to 88.32%, most works employed a classification approach and relied on data from contexts where stress was induced. Therefore, the primary aim of the present study was to clarify whether stress can be detected from facial expressions of six basic emotions (anxiety, anger, disgust, sadness, joy, love) and relaxation using a prediction approach.

**Method:**

To attain this goal, we analyzed video recordings of facial emotional expressions collected from n = 69 participants in a secondary analysis of a dataset from an interventional study. We aimed to explore associations with stress (assessed by the PSS-10 and a one-item stress measure).

**Results:**

Comparing two regression machine learning models [Random Forest (RF) and XGBoost], we found that facial emotional expressions were promising indicators of stress scores, with model fit being best when data from all six emotional facial expressions was used to train the model (one-item stress measure: MSE (XGB) = 2.31, MAE (XGB) = 1.32, MSE (RF) = 3.86, MAE (RF) = 1.69; PSS-10: MSE (XGB) = 25.65, MAE (XGB) = 4.16, MSE (RF) = 26.32, MAE (RF) = 4.14). XGBoost showed to be more reliable for prediction, with lower error for both training and test data.

**Discussion:**

The findings provide further evidence that non-invasive video recordings can complement standard objective and subjective markers of stress.

## 1 Theoretical background

It is late and you are preparing to leave the office. In the hallway, you meet your supervisor, and she asks you for an important report, which, as you suddenly remember, was due today. Your heart starts to race, you start to sweat, your stomach tightens: You are feeling stressed. Encountering a challenging situation such as this—experiencing a threatening situation while subjective coping resources are deemed insufficient—may prompt a stress response (1). This reaction is adaptive and necessary to ready an individual to either fight or flee the threat (2, 3). The stress response can be subdivided into the subjective experience of stress, typically assessed via self-report (e.g., the Perceived Stress Scale, PSS-10, 4), and the physiological stress response of nervous, endocrine, and immune mechanisms. Notably, both self-report and psychophysiological stress assessment suffer from important drawbacks. The assessment of subjective stress via questionnaire measures might be skewed due biases such as, e.g., social desirability (5) or extreme responding biases (6). Assessment of physiological markers, such as cortisol levels or heart rate, can be confounded by a plethora of factors, including sampling time, smoking, alcohol consumption, medication, physical activity levels, and use of hormonal contraception (7–9) and markers may not be specific to stress (10). Lastly, obtaining physiological measures might be perceived as obtrusive and their assessment and analysis requires considerable time and resources. Thus, they may not be feasible in every research context, which often limits physiological stress research to controlled (laboratory) situations such as the Trier Social Stress Test (TSST, 11). Thus, stress research could be advanced with scalable and accessible assessment tools.

Recently, novel technological developments have advanced non-invasive assessment of voluntary or involuntary behavioral changes occurring under stress and allow passive sensing of stress in various daily applications. In this context, tools already integrated into the everyday lives of many people, such as smartphones, could be used as sensors that are unobtrusive and easy-to-disseminate. Smartphones are typically equipped with a plethora of sensors that could potentially be used to measure stress (e.g., camera, depth sensors, gyroscope). Externally visible physiological changes under stress include both “macro” changes involving larger muscle groups (e.g., facial muscles, body posture) and “micro” changes, which are caused by physiological processes (e.g., Kurz et al., in preparation). As users typically face their smartphone when using it, and as facial expressions seem to a promising target of stress research (12, 13), the assessment of facial expressions via a smartphone front camera might be a promising avenue for stress assessment. Empirically, previous research on the relationship between stress and facial expressions found a link between arousal and visible facial expressions, as activity in several facial action units (AUs), which correspond to facial expressions, correlated with markers of the psychophysiological stress response (14). Another study found that reporting fear vs. indignation after a stress induction task was related to differential patterns in cortisol and cardiovascular activity (15). Lastly, one study found that not only confrontation with a stressor, but also anticipatory appraisal of a potentially stressful situation induced a cardiovascular stress response, highlighting how not only the presence of a “real” stressor but also our psychological appraisal can influence physiological responding (16). In a recent study (12, 13, 17, 18), we developed and evaluated a novel smartphone-based training to reduce stress by reacting to stress-related cognitions with facial expressions of positive and negative emotions, providing data on both emotional facial expressions during smartphone use and self-reported stress levels, which could be used to explore various emotional facial expressions as a marker for subjective stress.

In recent years, several researchers have successfully developed algorithms to detect stress based on video data of facial expressions, with accuracies of up to 88.32% (19–28). However, many of these studies use data collected in measurement setups in which stress was induced through performance demands (20, 23, 25, 27, 28) or in situations where individuals were prone to experience elevated stress, such as driving (21). Secondly, studies focused mostly on the role of negative emotions in stress prediction (22, 24, 26). Thirdly, the previously mentioned studies have employed classification instead of prediction approaches, which limits the applicability to real-world contexts, in which stress may change continuously and in nuanced patterns, and few studies validated their findings against a standard measure of stress. Lastly, research on the role of facial emotional expressions in the stress process has been hindered by the fact that detecting and labeling emotional expressions in video recordings by human raters, although reliable (29), requires intensive training and time (30). In recent years, software such as OpenFace2.0 (31) have harnessed machine learning (ML) approaches to allow for automatic detection of facial emotional expression in video data, extracting landmarks that can be used in further analyses using ML methods.

In summary, whereas these studies offer important insights in the role of facial expressions under stress, several gaps remain: Firstly, many studies experimentally induced high levels of stress and study its consequences. Less is known about correlates of subjectively reported stress in conditions where stress was not explicitly induced. Secondly, it is unclear which specific facial emotional expressions are associated with subjective stress and not only negative, but positive emotions should be considered. Individuals might not only experience stress in situations where they display negative emotions, but also when they display facial expressions of positive emotions. Different emotions with the same valence might be differentially associated with subjective stress levels (15). Lastly, current methods of detecting stress from facial expressions still often require extensive laboratory setups (32–36).

To address these gaps, this study analyzes videos of facial emotional expressions recorded via smartphone in a setting in which participants were prompted to display facial expressions of both negative and positive emotions (12, 13, 17, 18), but where stress was not directly induced. In the current study, we examined whether the extracted visual features processed with a Random Forest (RF) regression algorithm relate to subjectively reported stress.

## 2 Methods

This study is a secondary analysis of data from a randomized controlled pilot study which evaluated a smartphone-based intervention aimed to reduce stress. A detailed description of the study procedure can be found in the protocol paper (for the protocol paper, see 37). This study did not evaluate the efficacy and clinical potential of the intervention. The evaluation of the randomized controlled pilot study can be found elsewhere (13, 17, 18). The study was conducted in accordance with the declaration of Helsinki and ethical approval was obtained by the university's ethical review board. The study was preregistered in the German clinical trials register (Deutsches Register Klinischer Studien; DRKS00023007).

### 2.1 Participants

Individuals with elevated stress (*n* = 80) were recruited through advertisements in public places in Erlangen, a medium-sized German city. Participants were eligible to participate in the study if they (1) reported elevated levels of stress, corresponding to a score of 19 or higher in the German version of the Perceived Stress Scale-10 (PSS-10, 4, 38), (2) were ≥18 years, and (3) provided informed consent. Exclusion criteria were (1) acute severe psychiatric conditions/symptomatology (e.g., suicidal ideation, substance abuse, or psychotic symptoms), physical impairment of facial emotion expression (e.g., facial paralysis), and heavy smoking (due to the assessment of salivary cortisol, see protocol paper; 33). In this study, we analyzed data of *n* = 69 participants who were allocated to the experimental conditions, as only participants in these conditions were asked to display emotional facial expressions.

### 2.2 Procedure

Participants completed a 4-day training aimed to reduce stress. Stress was not specifically induced before the training, instead, participants were invited to work on their stress that arose from day-to-day life. The training required the display of various facial emotional expressions in response to written statements displayed on a smartphone screen. The statements included potentially stress-reducing beliefs (e.g., “It is okay to make mistakes.”) and stress-increasing beliefs (e.g., “I always have to be perfect.”). Participants were instructed to distance themselves from the stress-inducing beliefs by displaying negative facial emotional expressions (such as anxiety, anger, sadness, or disgust) and approach stress-reducing beliefs by displaying positive facial emotional expressions (such as joy, relaxation, confidence, or pride). Participants were randomly allocated to eight different intervention groups (six intervention and two control conditions). The six intervention groups differed in the negative facial emotional expressions participants were asked to display. Participants in the first group (*n* = 10) were asked to display anxiety in response to stress-inducing statements, the second group (*n* = 10) was asked to display anger, the third group (*n* = 10) was asked to display sadness, the fourth group (*n* = 10) was asked to display disgust, and the fifth and sixth groups (total *n* = 20) were asked to display all four negative emotions in varying ratios with the positive emotions (1:1 vs. 1:4). The emotions were chosen from the six basic emotions (39) and relaxation. Different experimental groups performed different negative emotions as the aim of the original study was to compare variations of the training enhanced by different emotions (37). Participants in all groups were asked to display the same positive facial emotional expressions (training day 1: joy, relaxation, and love; day 2: excitement, tranquility, and gratitude; day 3: happiness, resolve, and contentment; day 4: courage, confidence, and pride). The order of the emotions across days was determined before the beginning of the study to include a variety of different positive emotions. Participants were given examples on how the emotional expression could be performed by the experimenter and viewed videos of an actor displaying the different emotional expression on the study smartphone. The experimenter highlighted that these options should only serve as examples, and that the participant could perform the emotional expression as they would normally do (e.g., in terms of intensity, accompanying gestures, etc.). During the training session, participants' facial emotional expressions were recorded with a video camera and the experimenter provided feedback whether the expression had been performed correctly. To monitor participants' compliance with the instructions, the experimenter additionally rated the perceived quality of the facial emotional expressions in face and body. Participants in the active control condition (*n* = 10, not included in this study) were not specifically asked to display facial emotion expressions and participants in the inactive control condition (*n* = 10, not included in this study) did not participate in any laboratory intervention A full description of the study procedure can be found in the protocol paper (12).

### 2.3 Measures

A full overview of all outcome assessed in the study can be found in the protocol paper (12). This study included two measures of stress, the PSS-10 (4) and a one-item stress measure.

The PSS-10 (4) was assessed before the first training day (T1), after the last training day (T2), and 1 week after the last training day (T3). Participants rated statements regarding their stress level during the past week on a 5-point Likert scale (0 = *never* to 4 = *very often*). The German version of the PSS-10 has been found to have good reliability (Crohnbach's alpha = 0.84; 38). As the PSS-10 records stress experienced within the past week, it was included in this study to capture stress experienced over a longer period of time.

Stress at the time of assessment was assessed before the first study session and after the last study session with one self-constructed item using an 11-point Likert scale (0 = *not at all* to 10 = *very much*). The question stated (1) “Please indicate how stressed you feel at the moment” (current stress). This measure was included to capture participants' stress at the time of assessment. The item had a medium test-retest reliability, with a correlation of *r* = .29 between the two assessment time points.

### 2.4 Statistical analyses

#### 2.4.1 Facial emotion detection

Data on facial emotional expressions collected during the training session was used to explore associations with stress experienced over the past week (assessed with the PSS-10 at the beginning of the study) and with stress experienced at the time of assessment (assessed with the one-item stress measure at the beginning of the study).

To analyze facial emotional expressions, we employed the Facial Action Coding System (FACS), which provides a standardized taxonomy for observable and anatomically grounded movements of facial muscles (40). FACS assign a unique code to each AU, thereby enabling researchers to systematically analyze and classify facial expressions.

OpenFace2.0 (31) was utilized for extracting facial behavior features. This open-source toolkit enables real-time analysis of facial videos. Among the extracted parameters are (1) AUs, (2) facial landmarks, (3) eye gaze, and (4) head pose. OpenFace2.0 accurately predicts the presence and intensity of a subset of 18 AUs, namely AU1, AU2, AU4, AU5, AU6, AU7, AU9, AU10, AU12, AU14, AU15, AU17, AU20, AU23, AU25, AU26, AU28, and AU45). The intensity of an AU is defined on a 5-point scale (0 = *not present* to 4 = *present at maximum intensity*). The tool achieves an average Concordance Correlation Coefficient (CCC) of 0.73 on the DISFA dataset (31), indicating an agreement of 73% between the predicted AU intensities by OpenFace2.0 and manually annotated AU intensity in the DISFA dataset (41). Apart from AUs, facial landmarks also represent key facial features such as eyes, nose, mouth, and consist of 64 locations (2D and 3D) extracted per video frame. Along with these parameters, the angle of left and right eye gaze is given in radians. We analyzed the entire duration of the experiment using video recordings captured with a frame rate of 30 frames per second (fps). Additionally, the analysis incorporates the three-dimensional head position relative to the camera, in addition to rotational data: roll (rotation around the head's front-to-back axis), pitch (rotation around the head's side-to-side axis), and yaw (rotation around the head's vertical axis). This ensured comprehensive coverage of all emotional expressions exhibited by participants, rather than focusing only on specific movements.

In the next step, noise in the data was reduced in a four-step approach. Firstly, with the aid of the attribute “Confidence” in OpenFace 2.0, frames where less than 80% of the face were visible (e.g., during the start and end of the video where participants sometimes moved out of the recorded frame) were excluded. Secondly, only frames where the AU was 100% present were considered. We followed this approach as AUs are the primary features considered in this work and the AUs of interest might not be visible in all frames of the video if the participant had difficulties performing the emotional expression. Thirdly, the mean and standard deviation of the features across the video were computed to reduce noise and to capture the variability of emotional expression over the video. The missing values induced through the calculation of standard deviation were systematically eliminated p≤0.05.

#### 2.4.2 Training of machine learning models

To develop a stable model for detecting subjective stress scores from emotional facial expressions, we employed Random Forest (RF, 68) and Extreme Gradient Boosting (XGBoost, 37) as two ML-based regression techniques. The features derived from the video data served as input. These features were considered as the independent variables, whereas the stress score was considered as the dependent variable. The methodology followed a structured pipeline comprising feature standardization, hyperparameter tuning via cross-validation, model training using optimized parameters, and performance evaluation. Prior to training, we standardized the feature set to ensure that all input variables had a mean of zero and a standard deviation of one. As we used ensemble methods in our ML models, we included standardization as a preprocessing step to mitigate the impact of varying feature scales and enhances model stability. This transformation prevents features with larger magnitudes from disproportionately influencing the model, thereby ensuring a more balanced learning process.

To optimize the predictive performance of both models, we applied a hyperparameter tuning approach using randomized grid search within a five-fold cross-validation framework. Cross-validation ensures that the model generalizes well by evaluating it across multiple subsets of the data, thereby reducing the risk of overfitting. The tuning process was carried out in the following steps: Firstly, a predefined range of hyperparameters was established for both the RF and XGBoost models. Secondly, randomized grid search was conducted to sample hyperparameter combinations from the defined space, enabling efficient exploration of the parameter landscape without an exhaustive search. Thirdly, five-fold cross-validation was employed during the search process, splitting the dataset into five subsets where, in each iteration, the model was trained on four subsets and validated on the remaining one. Lastly, the best-performing hyperparameters were selected based on the lowest mean squared error (MSE) observed during cross-validation.

Once the optimal hyperparameters were identified, both the RF and XGBoost models were trained using five-fold cross-validation to ensure consistent performance across different data partitions. Prior to the model training, feature selection was performed using the SelectKBest method with an ANOVA *F*-test, retaining 750 most relevant features out of the original 1,315 features. This step not only enhanced model interpretability by focusing on the most informative features but also served as a dimensionality reduction technique, mitigating potential overfitting.

We first employed a RF regression algorithm, an ensemble learning method that enhances predictive accuracy and reduces overfitting (42). RF operates by constructing multiple decision trees during training and aggregating their predictions. This ensemble approach minimizes variance and improves generalization. The algorithm utilizes bootstrapping and bagging techniques, where bootstrapping generates diverse datasets by randomly sampling from the original data with replacement, and bagging ensures each tree is trained on a different subset of data. This de-correlation of data contributes to a more robust predictive model by mitigating overfitting. For this study, we employed the optimized RF regressor with the best-selected hyperparameters, ensuring that the model effectively learned the relationship between the facial features extracted from video data and the subjective stress scores of participants.

In addition to the RF model, we also trained an XGBoost regression model to predict subjective stress scores. XGBoost is an advanced gradient boosting framework that builds trees sequentially, where each new tree corrects the errors of its predecessors. Unlike traditional boosting methods, XGBoost incorporates advanced regularization techniques such as L1 and L2 regularization, which improve generalization and reduce the risk of overfitting. It also employs a weighted quantile sketch algorithm for efficient handling of sparse data and parallelized tree construction for computational efficiency. Moreover, XGBoost utilizes a unique split-finding algorithm that balances model complexity and predictive performance, making it well-suited for structured data applications. With these optimizations, the XGBoost model was trained using the optimized hyperparameters, following the same five-fold cross-validation process as the RF model.

#### 2.4.3 Model evaluation

The models were trained using the facial features extracted through OpenFace as input. The stress levels based on the PSS-10 and the one-item stress measure served as labels. For model evaluation, Mean Absolute Error (MAE) and Mean Squared Error (MSE) were used. MAE quantifies the average magnitude, while MSE emphasizes larger error due to squaring. Lower values for both metrices indicate better performance.

The mathematical representation of MAE and MSE are as follows:

$$\mathrm{MAE}=\frac{1}{n}\overset{n}{\underset{i=1}{\sum }}\;|y_{i}-{\hat{y}}_{i}|$$

Where:

$$\begin{array}{cc} n: & \mathrm{Number}\;\mathrm{of}\;\mathrm{data}\;\mathrm{points}\\ y_{i} & \mathrm{Actual}\;\mathrm{value}\;\mathrm{of}\;\;\mathrm{the}\;\mathrm{target}\;\;\mathrm{variable}\;\;\mathrm{for}\;\mathrm{the}\;i\mathrm{th}\;\;\mathrm{data}\;\;\mathrm{point}\\ {\hat{y}}_{i} & \mathrm{Predicted}\;\;\mathrm{value}\;\mathrm{of}\;\mathrm{the}\;\mathrm{target}\;\mathrm{variable}\;\mathrm{for}\;\mathrm{the}\;i\mathrm{th}\;\mathrm{data}\;\mathrm{point} \end{array}$$

$$\mathrm{MSE}=\frac{1}{n}\overset{n}{\underset{i=1}{\sum }}\;(y_{i}-{\hat{y}}_{i}{)}^{2}$$

Where:

$$\begin{array}{cc} n: & \mathrm{Number}\;\mathrm{of}\;\mathrm{data}\;\mathrm{points}\\ y_{i} & \mathrm{Actual}\;\mathrm{value}\;\mathrm{of}\;\;\mathrm{the}\;\mathrm{target}\;\;\mathrm{variable}\;\;\mathrm{for}\;\mathrm{the}\;i\mathrm{th}\;\;\mathrm{data}\;\;\mathrm{point}\\ {\hat{y}}_{i} & \mathrm{Predicted}\;\;\mathrm{value}\;\mathrm{of}\;\mathrm{the}\;\mathrm{target}\;\mathrm{variable}\;\mathrm{for}\;\mathrm{the}\;i\mathrm{th}\;\mathrm{data}\;\mathrm{point} \end{array}$$

MAE treats all errors equally due to its absolute nature and does not adequately penalize large errors or outliers. In contrast, MSE squares errors, thereby heavily penalizing large errors. In this study, both metrics were evaluated. The algorithm, however, tried to minimize MSE. The code that was used to generate and train the model is available under OSF.io (https://osf.io/ksyda/?view_only=5da173bacf4b485bb9e6e28510d3844b).

## 3 Results

### 3.1 Demographic characteristics

The demographic information for the sample is presented in Table 1. Overall, the sample was mostly female, highly educated, and young, with a mean age of 21.36 (SD = 16.65; range from 19 to 46). Descriptive statistics for PSS-10 and the one-item stress measure are presented in Table 2. Correlations between the PSS-10 and the one-item stress measure are displayed in Table 3. Neither baseline scores of the PSS-10 [*t*_(67)_ = −0.41, *p* = .682], nor of the one-item stress measure [*t*_(67)_ = 0.66, *p* = .514] differed significantly between male and female participants.

**Table 1:** Demographic characteristics of the sample (*n* = 69).

Variable	Frequency (percentage)
Gender	
Male	14 (20.29%)
Female	55 (79.71%)
Occupation	
Student	1 (1.44%)
University student	62 (89.86%)
Employee	6 (8.70%)

**Table 2:** Means (*M*), standard deviation (SD), minimum (min) and maximum (max) for PSS-10 scores and one-item stress measure at pre- and post-assessment (*n* = 69).

Variable	Pre			Post		
	*M* (SD)	Min	Max	*M* (SD)	Min	Max
PSS-10	20.17 (6.10)	5	33	15.72 (6.32)	0	31
Current stress (one-item measure)	4.17 (2.07)	0	8	3.51 (2.08)	0	9

**Table 3:** Pearson correlations between the PSS-10 and the one-item stress measure at pre- and post-assessment (*n* = 69).

Measures	Measures			
	PSS-10, t1	PSS-10, t2	One-item measure, t1	One-item measure, t2
PSS-10, t1	–	–	–	–
PSS-10, t2	.54	–	–	–
One-item measure, t1	.54	.17	–	–
One-item measure, t2	.34	.52	.29	–

### 3.2 ML model metrics

Table 4 illustrates the error metrics for the prediction of PSS-10 scores across considered emotions, while Table 5 presents the corresponding metrics for the prediction of the one-item stress measure. The MSE and MAE for PSS-10 predictions were 2.07 and 0.82, respectively, when all emotions are considered. For the one-item stress measure, the MSE and MAE were 0.41 and 0.35, respectively. The *R*^2^ score for PSS-10 prediction was 0.94 and that of one-item stress measure was 0.9 (Tables 4, 5).

**Table 5:** MSE and MAE error metrics for the RF and XGBoost algorithms for the training and test data sets for the one-item stress measure.

Emotion	Algorithm							
	XGB		XGB		RF		RF	
	MSE		MAE		MSE		MAE	
	Train	Test	Train	Test	Train	Test	Train	Test
Joy	2.40	2.92	1.32	1.46	2.89	4.01	1.43	1.72
Love	2.01	2.84	1.22	1.45	2.78	3.79	1.41	1.66
Relaxation	1.99	2.90	1.21	1.46	2.57	3.97	1.33	1.68
Anger	2.00	2.23	1.20	1.27	2.61	3.66	1.31	1.56
Disgust	2.03	2.88	1.09	1.50	2.09	3.36	1.24	1.57
Sadness	1.35	2.54	0.97	1.34	0.70	2.24	0.67	1.23
Anxiety	0.13	2.05	0.22	1.02	0.28	1.89	0.34	1.01
All emotions	2.12	2.31	1.26	1.32	3.45	3.86	1.56	1.69

MSE, mean squared error; MAE, mean absolute error.

**Table 4:** MSE and MAE error metrics for the RF and XGBoost algorithms for the training and test data sets for the PSS-10.

Emotion	Algorithm							
	RF		RF		XGBoost		XGBoost	
	MSE		MAE		MSE		MAE	
	Train	Test	Train	Test	Train	Test	Train	Test
Joy	19.17	24.94	3.65	4.14	23.73	32.77	4.01	4.76
Love	19.00	27.12	3.60	4.30	23.98	32.32	4.05	4.71
Relaxation	18.77	26.10	3.58	4.22	24.68	34.62	4.07	4.88
Anger	18.45	21.46	3.42	3.73	14.15	29.23	2.84	4.22
Disgust	19.77	21.53	3.73	3.87	13.53	15.95	2.75	2.97
Sadness	2.59	2.40	0.03	1.05	5.34	6.48	1.83	2.01
Anxiety	6.81	7.81	2.14	2.29	0.46	2.50	0.38	0.89
All emotions	25.10	25.65	4.12	4.16	25.83	26.32	4.10	4.14

MSE, mean squared error; MAE, mean absolute error.

## 4 Discussion

The aim of this exploratory study, conducted as a secondary analysis of data from a randomized controlled pilot study on a novel emotion-based intervention to reduce stress (12), was to assess how facial emotional expressions relate to stress. Previous studies have found the emotion-based intervention to be acceptable and clinically effective (13, 17, 18). Using video data collected during emotion-evoking smartphone use, we found that facial emotional expressions can be used to predict stress, as assessed using both the PSS-10 and a one-item stress measure, with low model error. The evaluation of XGBoost and RF algorithms across two different stress prediction tasks demonstrated marked differences their performance, generalization capability, and potential overfitting tendencies.

Overall XGBoost achieved lower MSE and MAE on the test set compared to RF, indicating higher robustness and better generalization to unseen data. This was particularly evident for the one-item stress measure, where XGBoost's average test MSE (2.31) was notably lower than RF's (3.86). Findings for PSS-10 were similar, as XGBoost maintained lower test errors, which indicates its advantage for handling structured tabular data with complex patterns. The performance advantage of XGBoost could be attributed to its gradient boosting approach, which sequentially corrects errors from previous iterations and enabling fine-tuned predictions. Additionally, XGBoost's built-in L1 and L2 regularization mechanisms helped control overfitting and ensure stable generalization. In contrast, RF exhibited higher test errors (particularly for MSE) which indicated a tendency to overfit. The larger gap between training and test errors in RF suggests that its reliance on bagging, which reduces variance but does not explicitly refine errors iteratively, might lead to suboptimal generalization compared to boosting techniques.

An important difference between the two algorithms was found for their performance on data from different emotional expressions: For positive emotions such as joy, love, and relaxation, and higher stress levels in PSS-10, both models showed similar performance trends. However, RF exhibited a larger discrepancy between training and test errors, indicating mild overfitting. The improvement for test MSE with XGBoost (e.g., 2.92 for joy vs. 4.01 for RF) indicates that it can capture emotional patterns without overfitting. For anger and disgust, XGBoost significantly outperformed RF, with the largest performance gap observed in disgust (RF test MSE: 3.36 vs. XGBoost test MSE: 2.88). This suggests that RF might struggle with generalizing lower-intensity or nuanced emotional expressions, potentially due to noise sensitivity in bagging-based approaches. For the prediction of anxiety, the overfitting issue in RF was most pronounced (train MSE: 0.28, test MSE: 1.89). Similarly, train MSE values were significantly lower than test MSE values for PSS-10, which indicates that RF memorized the training data rather than learning generalizable patterns. In contrast, XGBoost maintained a more balanced train-test error ratio, which indicates that it can capture complex stress patterns.

Another important finding was that both models had important performance differences when aiming to detect subtle changes in stress. In general, and particularly for higher PSS-10 scores, XGBoost showed consistent test errors across multiple runs, while RF exhibited fluctuating test performance. This shows that boosting techniques like XGBoost are more stable for analyzing psychological stress prediction. Similarly, RF exhibited overfitting tendencies for lower stress in PSS-10, with an increase in test errors compared to training errors. XGBoost had a more stable train-test error relationship, which might make it more suitable to detect subtle psychological patterns. Taken together, these findings highlight the potential applicability of XGBoost-based automated emotion and stress recognition systems for psychology, where reliability, stability, and generalization are paramount.

From a psychological perspective, several study design features might explain these findings. Firstly, in line with the hypothesis that both stress and emotion are linked through arousal and appraisal processes (43, 44), anger, disgust, and sadness might have been characterized by higher arousal and the activation of more AUs (45), which might have increased the number of parameters that could be extracted in OpenFace and used as the basis for model evaluation. In line, emerging literature on changes in facial emotional expressions under stress indicates an association between stress and emotions such as anger (46) and activity in the musculus corrugator supercilii (47), which is associated with negative facial emotional expressions (42, 48). In turn, positive emotional expressions (joy, love, relaxation) might have been characterized by weaker facial muscle activity, making them more difficult to detect with OpenFace. Secondly, it should be noted that there was a gender imbalance of the sample, with 80% of participants being female. However, males and females have been only found to differ with regard to the frequency with which different emotions are displayed and not how they are displayed (49–52). As all participants in the current study were instructed to perform the same emotional expressions and there were no significant differences in baseline stress, it is unlikely that the gender imbalance may have impacted the results. Lastly, despite being routinely used as a measure of stress in many studies (53), the accuracy for models including stress assessed with the PSS-10 (4) was slightly lower than that for the one-item stress measure. Whereas the PSS-10 asked participants to rate their stress experienced during the past week, the one-item measure asked participants to indicate their current stress at the time of assessment. Thus, the response in the one-item stress measure might have more closely reflected participants' stress at the time of video data collection (54–57).

This study yields several important implications: Firstly, this study is one of the first to use a prediction instead of a classification algorithm. Previous studies (21–23, 25–27) have used classification approaches to distinguish stressed from non-stressed individuals using ML approaches. However, instruments such as the PSS-10 (4) capture subjective stress on a continuous scale, making prediction approaches more suitable for capturing nuanced changes in stress. Secondly, this study shows that that stress detection using ML-based analysis of video data is also possible in contexts in which stress was not directly induced (albeit individuals may have experienced some degree of stress due to the study context), contrasting previous studies that used data from contexts in which stress was directly induced through performance demands or individuals were likely to experience high levels of subjective stress (see e.g., 21, 22) or have used standardized laboratory paradigms such as the TSST (11).

Although this study has shed light on potential new methods of stress detection, there are several important limitations to be considered: Firstly, we predicted stress based on a pre-specified set of positive and negative facial emotional expressions instead of spontaneously expressed emotions and subjective emotion intensity was not assessed. Furthermore, we did not ask participants how strongly they experienced the emotion in question. Additionally, individuals may differ in their facial expressiveness (e.g., due to differences in cultural background, emotion regulation, and personality structure), which might impair prediction accuracy for individuals with low expressiveness and overall model generalizability. Fourthly, we used a one-item rating to assess momentary stress over the course of the study. While this is a valid and frequent procedure in longitudinal psychological studies (54), the measure was not previously validated. Lastly, the sample size was relatively small, and the sample was not representative of the general population, limiting the generalizability of the findings.

From these limitations, several directions for future research can be derived: Firstly, further investigation into hybrid models combining RF and XGBoost could be beneficial, leveraging the variance reduction of bagging (RF) and sequential refinement of boosting (XGBoost). Additionally, feature engineering techniques such as interaction terms, domain-specific transformations, and deep feature selection could further enhance model accuracy. Secondly, the unobtrusive, video-based measurement setup used in the current study could complement widely used, but often intrusive and distracting measurement paradigms employed in clinical psychological research and practice. A next step in research could be to connect these existing methods (55, 56) to develop minimally invasive measurement set-ups. Sensor fusion approaches could leverage input from various sensors, collecting data on different physiological systems to develop multi-modal stress detection systems and explore whether including physiological parameters such as heart rate, voice, and respiratory activity can improve prediction accuracy. These approaches should be evaluated in real-time scenarios, as in-the-moment stress detection using smartphone data could allow the delivery of just-in-time interventions (57–59) to target stress as it emerges. Samples should be representative of the general population to avoid biases and allow for higher generalizability of the results. In this context, researchers should specifically explore potential differences between genders (49, 50, 60) and individuals from various cultural backgrounds. Additionally, studies should also consider the role of positive emotions, as researchers have long argued that positive emotions may also serve important functions in the stress process (61) preliminary evidence suggests that deliberately showing expressions of positive emotions can decrease the detrimental effects of stress (62). Using ML models, a previous study was able to successfully distinguish self-reported distress from eustress (63). Future works should consider the impact of positive stress-related emotional experiences and distinguish between eustress and distress states. Finally, as these methods allow for unobtrusive measurement of inner states, research in this context should be accompanied by ethical considerations to ensure that measurement setups comply with ethical standards (64, 65). In ML contexts, special care should be taken to ensure that the resulting model is free of biases (66, 67), highlighting the need for appropriate data samples (in terms of gender, ethnicity, and socioeconomic background). Additionally, stress is a sensitive psychological state, so stress detection models should comply with data privacy laws and protect the healthcare information of their users.

As an interdisciplinary study connecting biopsychological stress research and ML methods, this study highlights how an this perspective can advance psychophysiological stress research. Using contactless approaches to detect indicators of inner states (such as macro- or even micro movements) might offer novel ways of measuring psychological processes.

## 5 Conclusion

This study aimed to explore whether macro movements in the form of emotional facial expressions recorded on video can be correlated with subjectively experienced stress. Using both a RF and XGBoost algorithm, we found that overall model accuracy for stress scores was good, with model accuracy being better for negative facial emotional expressions. XGBoost demonstrated better generalization, particularly for subtle patterns in emotional intensity and stress levels. This makes it a more reliable choice for real-world applications where unseen data distributions must be handled effectively. RF exhibited higher overfitting tendencies, particularly in lower-intensity emotions (e.g., disgust) and complex psychological states (e.g., anxiety, PSS-10 scores). Additional regularization strategies or hybrid approaches may be required to enhance its generalization. By demonstrating that stress can be inferred from facial emotional expressions, this study further contributes to the emerging field of research on non-invasive methods to detect inner states and offers important opportunities for further research to improve diagnostic methods in psychology.

## Data Availability

The raw data supporting the conclusions of this article will be made available by the authors, without undue reservation.
